# Hemispherical acoustic Luneburg lens with the acoustic Goos–Hänchen shift and Fresnel filtering effect

**DOI:** 10.1038/s41598-020-76111-4

**Published:** 2020-11-04

**Authors:** Choon Mahn Park, Geo-Su Yim, Kyuman Cho, Sang Hun Lee

**Affiliations:** 1 Department of Materials Physics, Dong-A University, Busan, 49315 South Korea; 2 Department of Electrical Engineering, Pai Chai University, Daejeon, 35345 South Korea; 3 Department of Physics, Sogang University, Seoul, 04107 South Korea

**Keywords:** Acoustics, Metamaterials

## Abstract

A two-dimensional (2D) slice of a 3D hemispherical acoustic Luneburg lens using a quasi-conformal transformation and face-centred-orifice-cubic (FCOC) unit cells is designed and fabricated. With the system, the focusing characteristics of acoustic waves with frequencies that satisfy the homogeneous medium condition of the metamaterial are observed, such as focusing of acoustic plane waves at the antipodal point on the transformed surface of the opposite side for the incident direction and focus spreading due to total internal reflection at the focus point. The attenuation losses of the system are measured and compared with those of an untransformed system with respect to frequency. The value of the acoustic Goos–Hänchen shift is determined by comparing the experimental and theoretical and simulated values of the focus points with respect to the incident angle. The effect of acoustic Fresnel filtering due to the angular distribution of the incident waves at the flat surface boundary is verified by comparing the results of the experiment and a simulation.

## Introduction

Phenomena with a metamaterial that has both negative permittivity and permeability were studied by the Russian scientist Veselago in 1967^1^. Thirty years later, Smith et al. showed an experimental result for a metamaterial with a negative refractive index^2^. Since then, there have been studies of physical phenomena in various areas, such as negative and zero refractive index values^3,4^. In particular, it has attracted much attention because it can be used to actually implement an application such as a transparent cloak or concealment^5,6^. Additionally, in this field, the range of applications is very wide, depending on whether the wave used is an electromagnetic wave or a sound wave^7^.

In the case of electromagnetic waves, the refractive index of the material is defined as the ratio of the velocity of the electromagnetic wave in vacuum to that of the medium therein. The refractive index value of a material is greater than one, because the velocity in the material is smaller than that in vacuum. However, in the case of sound waves, the acoustic refractive index is defined as the ratio of the sound velocity in air to that in the medium. The value of the acoustic refractive index of a material is less than one, since the sound velocity in the material is generally faster than that in air. If the acoustic refractive index of a unit cell is greater than one like the optical refractive index, it is possible to realize an acoustic device that has the same shape and characteristics as an optical device. Additionally, the results of a study of the geometrical optical properties can also be similarly applied to geometrical acoustics. Therefore, to achieve a refractive index greater than one, we have to ensure that the velocity of the sound wave is slow in the material.

We observed that an orifice plate inserted in a cylindrical waveguide reduces the velocity of a sound wave in the waveguide. A two-dimensional (2D) face-centred-orifice-cubic (FCOC) unit cell, which is a kind of orifice-type unit cell with an acoustic refractive index greater than one, was designed^8^. The unit cell may be considered as a cross-superposition of one-directional unit cells and as a complementary cell of the face-centred-cubic (FCC) unit cell. Using FCOC unit cells, we successfully realized acoustic convex, GRIN and Luneberg lenses^8–10^. The FCOC unit cells used in convex lenses have symmetrical orifice diameters in all directions and, in the case of GRIN lenses, 1D asymmetry. In the case of the Luneberg lens, there is a characteristic that all have asymmetry in each direction. It can be seen that such acoustic lenses correspond almost identically to optical lenses and their associated characteristic equations^11,12^.

The Luneberg lens has the characteristic that the focal point of the waves is formed at the antipodal point of the incident direction on the surface of the device^13^. Therefore, it is possible to effectively observe phenomena that exceed the classical diffraction limit due to the near-field effect, such as evanescent waves or super lensing effects observed mainly near the surface^10,14–18^. Meanwhile, there have been various attempts to implement acoustic devices with new functionality by controlling acoustic beams^19,20^. Among these attempts, the conformal transformation method can be used to reshape the form of the structure while maintaining its physical characteristics, so much research is being carried out in this area^13,21–24^. In particular, in a flattened Luneburg lens, which is properly and conformally transformed, a 2D flat detector array can be used to observe the focused image, since the focus is formed on the flat plane. In addition, it is possible to make sure of the acoustic Goos–Hänchen shift (GHS) by the total internal reflection and the acoustic Fresnel filtering effect (FFE) due to the angular distribution of the acoustic wave incidence angle^25–28^. Acoustic GHS is a must take into account when designing an accurate acoustic lens, and acoustic FFE is an important factor in accurately predicting the propagation of sound waves after passing through an acoustic device.

In this paper, we realized a flattened hemispherical acoustic Luneburg lens, which consists of FCOC unit cells, using the conformal transformation result^22^. From an experiment and a simulation, we observed, for the first time, the effect of the acoustic GHS and FFE of the device.

## Theoretical description

The major characteristic of a spherical Luneburg lens is the focusing of waves at the antipodal point of the incident direction. If the sphere of the lens is transformed to a hemisphere, the transformed focusing surface will be a flat plane. To obtain the transformed electric permittivity tensor of the hemispherical Luneburg lens, we have to consider the Jacobian transformation matrix first as shown in Eq. (1).

1 $$\begin{aligned} \Lambda ^{i'}_{i} ={\frac{\partial {x_i}'}{\partial {x_i}}} = \left( \begin{array}{ccc} {\frac{\partial {x_1}'}{\partial {x_1}}}&{}{\frac{\partial {x_1}'}{\partial {x_2}}}&{}{\frac{\partial {x_1}'}{\partial {x_3}}} \\ {\frac{\partial {x_2}'}{\partial {x_1}}}&{}{\frac{\partial {x_2}'}{\partial {x_2}}}&{}{\frac{\partial {x_2}'}{\partial {x_3}}} \\ {\frac{\partial {x_3}'}{\partial {x_1}}}&{}{\frac{\partial {x_3}'}{\partial {x_2}}}&{}{\frac{\partial {x_3}'}{\partial {x_3}}} \end{array} \right) , \end{aligned}$$

where $x_i$ and $x_{i'}$ are the coordinate components in the untransformed and transformed domains, respectively. The electric permittivity tensor in the transformed system $\varepsilon ^{\hat{i'}\hat{j'}}$ can be written as

2 $$\begin{aligned} \varepsilon ^{{\hat{i'}}{\hat{j'}}}=(\text{det} \Lambda _{\hat{i}}^{\hat{i'}})^{-1} \Lambda _{{\hat{i}}}^{\hat{i'}} \Lambda _{{\hat{j}}}^{\hat{j'}} \varepsilon ^{{{\hat{i}}}{\hat{j}}}, \quad \Lambda _{\hat{i}}^{\hat{i'}}= \Lambda _{i'}^{\hat{i'}} \Lambda _{i}^{i'} \Lambda _{\hat{i}}^{i}. \end{aligned}$$

$\Lambda _{i'}^{\hat{i'}}$ represents the transformation from the coordinate basis $i'$ to the unit basis $\hat{i'}$ of the primed (or transformed) system. After the transformation of the sphere to the hemisphere with the coordinate of $(\rho {^{'}}, \phi {^{'}}, z^{'})$ and diagonalization with $(\rho {^{''}}, \phi {^{''}}, z^{''})$, the transformed electric permittivity and the refractive index can be obtained. If we set the magnetic permeability tensor $\mu ^{{\hat{i}}{\hat{j}}}$ as the identity tensor for a Luneburg lens with the refractive index of the surrounding air $n_0$, the electric permittivity tensor $\varepsilon ^{{\hat{i}}{\hat{j}}}$ can be written as follows^13^:

3 $$\begin{aligned} \mu ^{\hat{i}\hat{j}}=1, \; \varepsilon ^{\hat{i}\hat{j}} = \; n_L^2 (r) \; \delta ^{\hat{i} \hat{j}}\hbox { and }\, n_L (r)= n_0 \; \sqrt{(2-r^2/a^2)}. \end{aligned}$$

The electric permittivity of the hemispherical Luneburg lens is derived as follows^22^:

4 $$\begin{aligned} \varepsilon ^{{\hat{i''}}{\hat{j''}}} = \left( \begin{array}{ccc} {\mu _{+}}&{}{0}&{}{0} \\ {0}&{}{2}&{}{0} \\ {0}&{}{0}&{}{\mu _{-}} \end{array} \right) \varepsilon ^{{\hat{i}}{\hat{j}}} = n_L^2 (r) \left( \begin{array}{ccc} {\mu _{+}}&{}{0}&{}{0} \\ {0}&{}{2}&{}{0} \\ {0}&{}{0}&{}{\mu _{-}} \end{array} \right) =\left( \begin{array}{ccc} {n_{11}^2}&{}{0}&{}{0} \\ {0}&{}{n_{22}^2}&{}{0} \\ {0}&{}{0}&{}{n_{33}^2} \end{array} \right) , \end{aligned}$$

where

5 $$\begin{aligned} \mu _{+} = \left( 1 + \frac{1}{4(1-t^2)} \right) + 3 \frac{\sqrt{(1-t^2)+(\frac{t}{3})^2}}{4(1-t^2)}, \; \mu _{-} = \left( 1 + \frac{1}{4(1-t^2)} \right) - 3 \frac{\sqrt{(1-t^2)+(\frac{t}{3})^2}}{4(1-t^2)}, \quad t=\frac{\rho '}{a}. \end{aligned}$$

The values of $\mu _{+}$ and $\mu _{-}$ are greater than 2 and less than 1/2, respectively. $\rho {^{'}}$ and *a* are the radial distance from the $z'$ axis and the radius of the Luneburg lens, respectively. The result is applied to the acoustics. To realize the transformed hemispherical acoustic Luneburg lens, we use FCOC unit cells with different orifice diameters with respect to their positions using Eq. (6).

**Figure 1:**
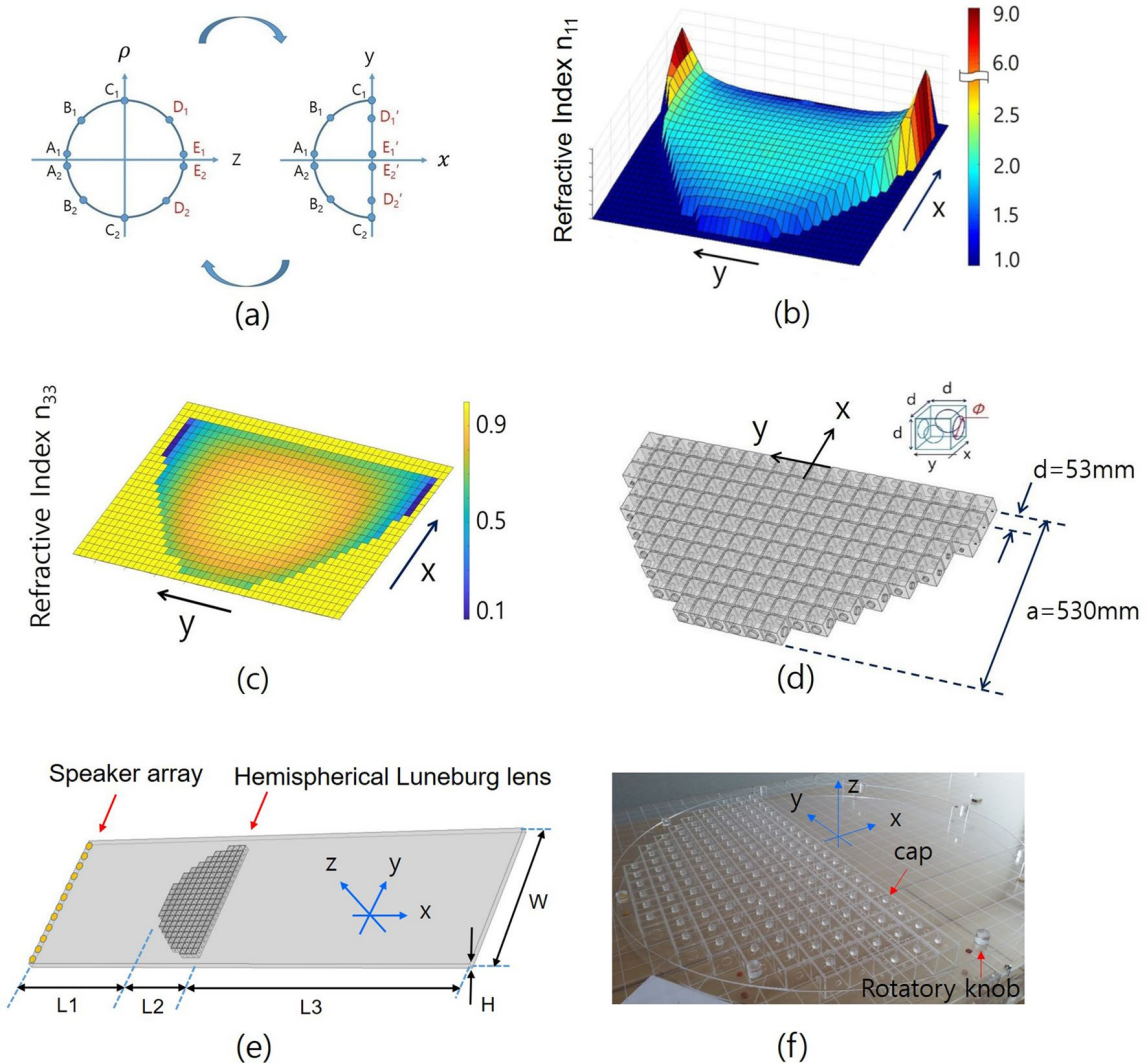
(**a**) shows the transformation pair between the sphere and hemisphere. The points $D_1, E_1, E_2,$ and $D_2$ on the right surface of the sphere are transformed to the points $D_1', E_1', E_2',$ and $D_2'$ on the right flat plane of the transformed hemisphere, respectively. (**b**,**c**) show the spatial distributions of the refractive index $n_{11}$ and $n_{33}$, respectively. (**d**) shows the designed 2D hemispherical Luneburg lens with FCOC unit cells using the approximation $n_{33} \simeq n_{11}$. (**e**,**f**) show a schematic diagram of the experimental setup and photograph of the hemispherical Luneburg lens fabricated, respectively. In the (**f**), a cap is for measuring the sound pressure inside the FCOC unit cell, and a rotatory knob is to handle for giving the angle of incidence. The lengths of L1, L2, L3, W and H are 1070, 530, 1920, 1150 and 50 mm respectively.

Figure 1a shows that the points *D*1, *E*1, *E*2,  and *D*2 on the right surface of the sphere are transformed to the points $D1', E1', E2',$ and $D2'$ on the right flat plane of the transformed hemisphere, respectively. The values of $n_{11}$ are greater than one, and the values of $n_{33}$ are less than one.

## Design and fabrication of the experimental setup

We use the approximation that the values of $n_{33}$ are neglected and approximately equal to those of $n_{11}$, because sound waves with $n_{33}$ diverge given that the refractive index is less than one^29^. Additionally, since the sound velocity for $n_{33}$ is faster than that for $n_{11}$, the divergence effect of $n_{33}$ is associated with an earlier time than $n_{11}$, which contributes to the focusing effect. Therefore, the term $n_{33}$ does not contribute to the focusing behavior of the device at the same time. Instead, we approximate that the values of $n_{33}$ are nearly equal to the values of $n_{11}$ to increase the symmetry and isotropicity of the FCOC unit cell in arbitrary positions of the device for the sound pressure amplitude^30^. When we set the values of $n_{33}$ to one, in the simulation, we observe that the smaller the angle of incidence is, the smaller the expected focusing effect. In the case of normal incidence, most waves are transmitted as if there is no lens, and focusing is not observed. This is because the refraction effect of the unit cell for sound waves that satisfy the homogeneous medium condition is very small when the incident angle is zero.

We fabricated a transformed hemispherical acoustic Luneburg lens consisting of FCOC unit cells, as shown in Fig. 1d. The volume of the cavity of the unit cell is 125 cm$^3$; the orifices that form the sidewalls of the cell were created by drilling a hole in the centre of a 3.0-mm-thick acrylic plate with a diameter calculated using Eqs. (4)–(6)^10,22^.

6 $$\begin{aligned} n_{eff} = n_{o} [1 + ( t' / d ) (S_w- S_{or})/S_{or} ]^{1/2}, \quad n_o = \sqrt{(\rho _0 / B_0)}. \end{aligned}$$

$t'$ is the effective thickness, *d* is the unit cell length, and $S_w$ and $S_{or}$ are the cross-sectional areas of the waveguide and the orifice, respectively. The centre axis of the device is now set as the x-axis, and the device has y-symmetry, as shown in Fig. 1d. Figure 1b shows the spatial distribution of the refractive index of the transformed hemispherical Luneburg lens $n_{11}$. The maximum and minimum values of $n_{11}$ are 9.547 at both side ends and 1.345 at both the front and back regions of the device. Figure 1c shows the spatial distribution of $n_{33}$, over which the values are distributed between 0.9977 and 0.1053. Figure 1d shows the designed 2D hemispherical Luneburg lens with the FCOC unit cells, where we have used the approximation $n_{33} \simeq n_{11}$. The diameter and height of the device are 1060 and 50 mm, respectively. The inset indicates the FCOC unit cell with an orifice diameter ${\phi }$, thickness *t* (3 mm), and unit cell length *d* (53 mm). The 2D FCOC unit cell can be regarded as a cross-superposition of 1D orifice-type unit cells of the x and y directions. The maximum and minimum values of the orifice diameter are 29.8 and 1.6 mm, respectively, and correspond to the minimum and maximum values of the refractive index $n_{11}$, respectively.

Rectangular waveguides are installed to guide the incident and transmitted waves on the left- and right-hand sides of the Luneburg lens. The dimensions of these waveguides are the same on both sides: a width of 1150 mm, a height of 50 mm, and a thickness of 3 mm. The propagation length for the incident waves is 1070 mm, from the speaker array acting as the sound source to the left-hand side vertex of the hemispherical 2D lens, while the propagation length for the transmitted waves is 1920 mm, from the transformed flattened surface of the hemispherical lens to the end boundary. Figure 1e shows a schematic diagram of the experimental setup. A photograph of the fabricated hemispherical acoustic Luneburg lens with FCOC unit cells is shown in Fig. 1f.

## Experiment and methods

To generate acoustic plane waves, a 12-speaker array with a length of 1150 mm is located at the left-hand end boundary of the waveguide as shown in Fig. 1e. The acoustic plane waves generated by this sound source enter the vertex of the transformed hemispherical Luneburg lens for normal incidence. To change the direction of the incidence, we turn the device (or the perpendicular axis of the device plane) around counterclockwise. Because of the frequency constraint of the homogeneous medium condition for the metamaterial (i.e., $d < \lambda /4$) and the value of the attenuation loss of the system, the highest and lowest frequencies for the employed metamaterial are approximately 1150 Hz ($d \simeq \lambda /6$) and 350 Hz ($d \simeq \lambda /20$), respectively, where the value of the sound velocity is 345 m/s^31^. Amplitude-shift-keying modulated acoustic pulses with a frequency of 2 Hz and a width of 10 ms are used to eliminate the echoes stemming from the multiple reflections that occur at the boundaries^32–34^. These acoustic pulses are activated simultaneously and in-phase using a functional generator. A sound absorber is used on the right-hand end of the waveguide to minimize any reflected waves and is experimentally confirmed to operate in our required frequency range. To obtain a perfect description of the wave propagation, the pressure amplitudes and the retarded times from the sound source are measured at 53 mm intervals along the x and y directions throughout the whole system, including inside and outside the hemispherical Luneburg lens, using a condenser-type microphone. To increase the reliability of the measurement, the average values of the pressure amplitudes and the retarded times for the sound waves are obtained by measuring the acoustic signals ten times at each position.

## Results and discussion

**Figure 2:**
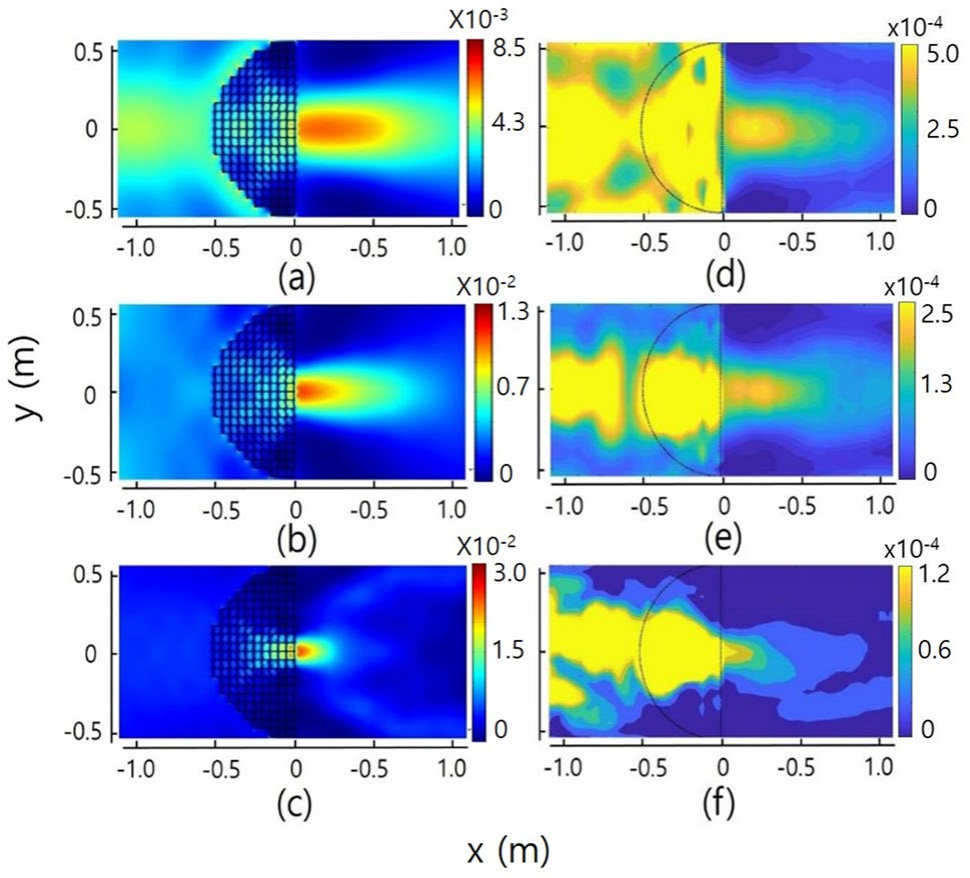
Intensity distributions of the acoustic waves after passing through the transformed hemispherical Luneburg lens for normal incidence. (**a**–**c**) show the simulation results for frequencies of 450, 650, and 1050 Hz, respectively, where the attenuation losses are not considered. (**d**–**f**) are the experimental results corresponding to (**a**–**c**), respectively. The intensity range of each color bar of the figures has arbitrary values in each figure.

Figure 2 shows the intensity distributions of the acoustic waves after transmittance through the transformed Luneburg lens, where the directions of incidence of the waves are all parallel to the centre axis (or x-axis). Figure 2a–c show the simulation results for wave frequencies of 450, 650, and 1050 Hz, respectively, where the attenuation losses are not considered. Figure 2d–f are the experimental results corresponding to Fig. 2a–c, respectively. By comparing the ratio of the maximum intensity of the incident region to that of the focused region in the experiment with that in the simulation, we determined the attenuation losses of the transformed hemispherical Luneburg lens with respect to the frequencies. The values of the attenuation loss in Fig. 2d–f are 0.7, 0.92, and 0.98 Np/m, respectively, where the intensity range of each color bar of the figures has arbitrary values in each figure. The values of attenuation loss are approximately 6–7 times larger than those of the untransformed acoustic Luneburg lens^10^. This result is reasonable, because the values of the distribution of the orifice diameter for the transformed hemispherical Luneburg lens are smaller than those for the untransformed spherical Luneburg lens. As the orifice diameter decreases, both the refractive index and the extinction index increase^9^. In the case of the hemispherical Luneburg lens, the values of the orifice diameter are distributed in the range of 1.6 to 29.8 mm, whereas in the case of the spherical Luneburg lens, the values are distributed in the range of 26.6 to 48 mm^10^. We determined the effective reflectivity $r (=\text{P}_{\text{ref}}/\text{P}_{\text{inc}})$ of the waves for the device by measuring the value of the standing wave ratio (SWR) along the centre axis with the frequencies. The measured values of the effective reflectivity are 0.18, 0.29, 0.25 and 0.21 at frequencies of 450, 650, 850 and 1050 Hz, respectively. From the results, we verify that the effective power reflectance of acoustic waves for the transformed hemispherical Luneburg lens is less than $\sim 8.5$ % of the incident power in the frequency region.

**Figure 3:**
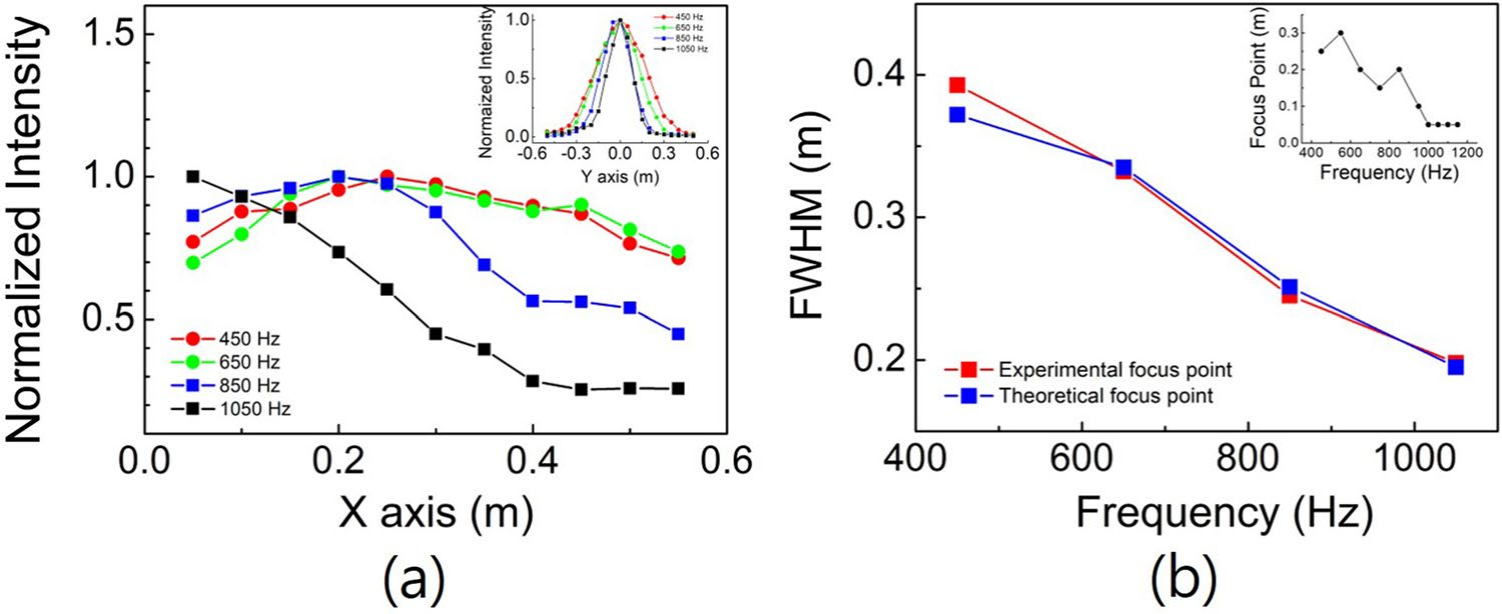
(**a**) shows the normalized intensity distributions measured along the center axis including the focus points at the given frequencies of 450 (red circle), 650 (green circle), 850 (blue square) and 1050 (black square) Hz, respectively, for normal incidence. The inset shows the normalized intensity distributions measured along the transverse direction (or y-axis) for the propagation including the experimental focus point at the same frequencies above. (**b**) shows the FWHM values of the acoustic intensity spots at the focused points of the centre axis with respect to frequency. The red squares show the values measured at the experimental focus point of the intensity spot, and the blue squares indicate the values measured at the theoretical focus point. The inset indicates the change in the distance of the focus point from the centre of the flattened surface along the centre axis with respect to frequency.

Figure 3a shows the normalized intensity distributions measured along the centre axis including the focus (or the maximum intensity) points at the given frequencies of 450 (red circle), 650 (green circle), 850 (blue square) and 1050 (black square) Hz, where the incident directions of the waves are all parallel to the centre axis. The inset shows the normalized intensity distributions measured along the transverse direction (or y-axis) for the propagation including the experimental focus (or maximum valued) point at the same frequencies above. Figure 3b shows the full width at half maximum (FWHM) values of the acoustic intensity spots at the focused points on the centre axis with respect to the frequencies. The red squares show the values measured at the experimental focus (or maximum valued) point of the intensity spot, and the blue squares indicate the values measured at the theoretical focus point (or at the point on the transformed flattened surface). We measured the sound pressure of the theoretical focus at a distance of 50 mm perpendicular to the flat surface of the device to avoid the edge effect of the unit cell orifice. Since these two graphs are nearly identical, we confirm that the acoustic waves are focused almost at the transformed flattened surface (i.e., theoretical focus plane) after passing through the lens. The upper right inset indicates the change in the distance of the focus point from the centre of the flattened surface along the centre axis with respect to frequency. At low frequencies, the focus point is slightly off the surface. With increasing frequency, the focus point becomes close to the surface. This is due to the divergence effect of the waves with respect to frequency.

**Figure 4:**
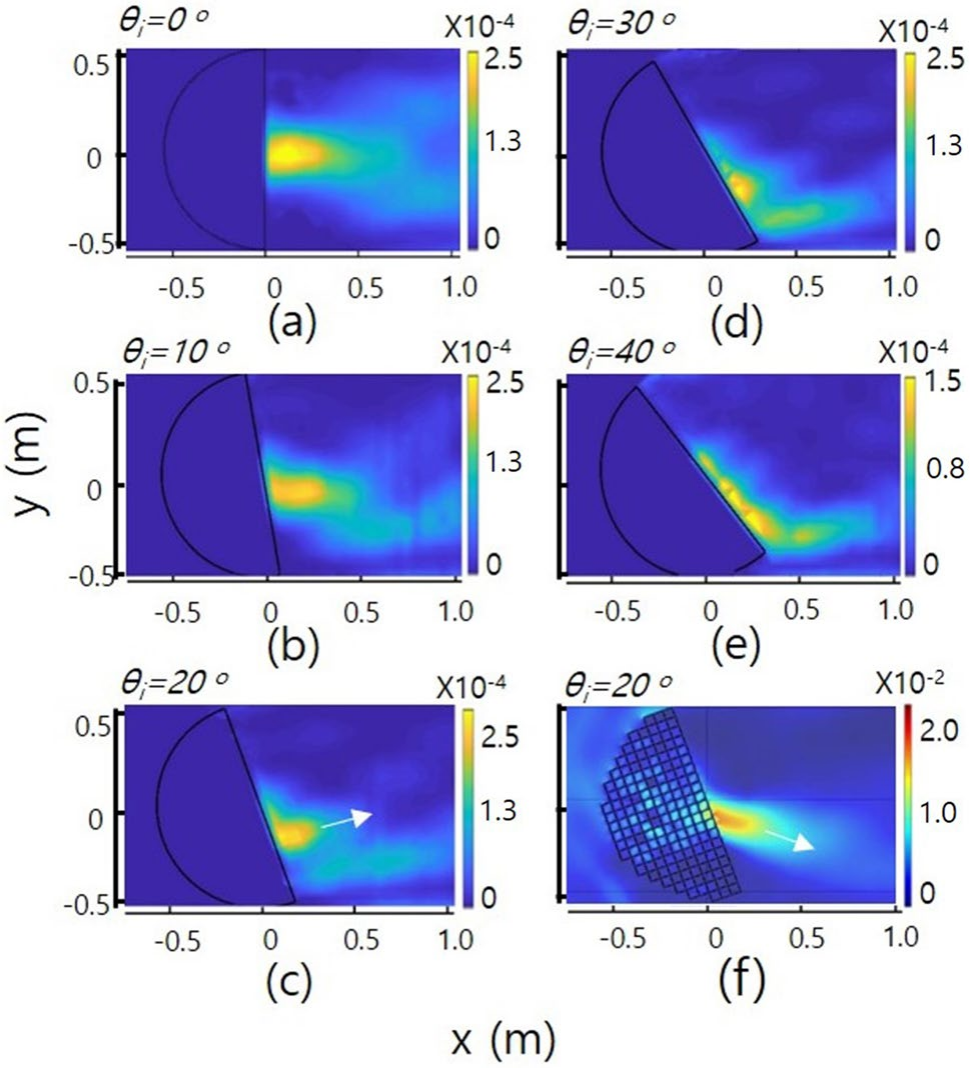
(**a**–**e**) show the experimental results of focusing of acoustic waves with a frequency of 850 Hz after passing through the device when the incident angles of the waves are 0, 10, 20, 30, and $40^{\circ }$, respectively. (**f**) shows the simulation result when the incident angle is $20^{\circ }$ for a comparison with (**c**). The white arrows in the figures show the directions of the acoustic radiation after passing through the flattened surface. The intensity range of each color bar of the figures has arbitrary values in each figure.

**Figure 5:**
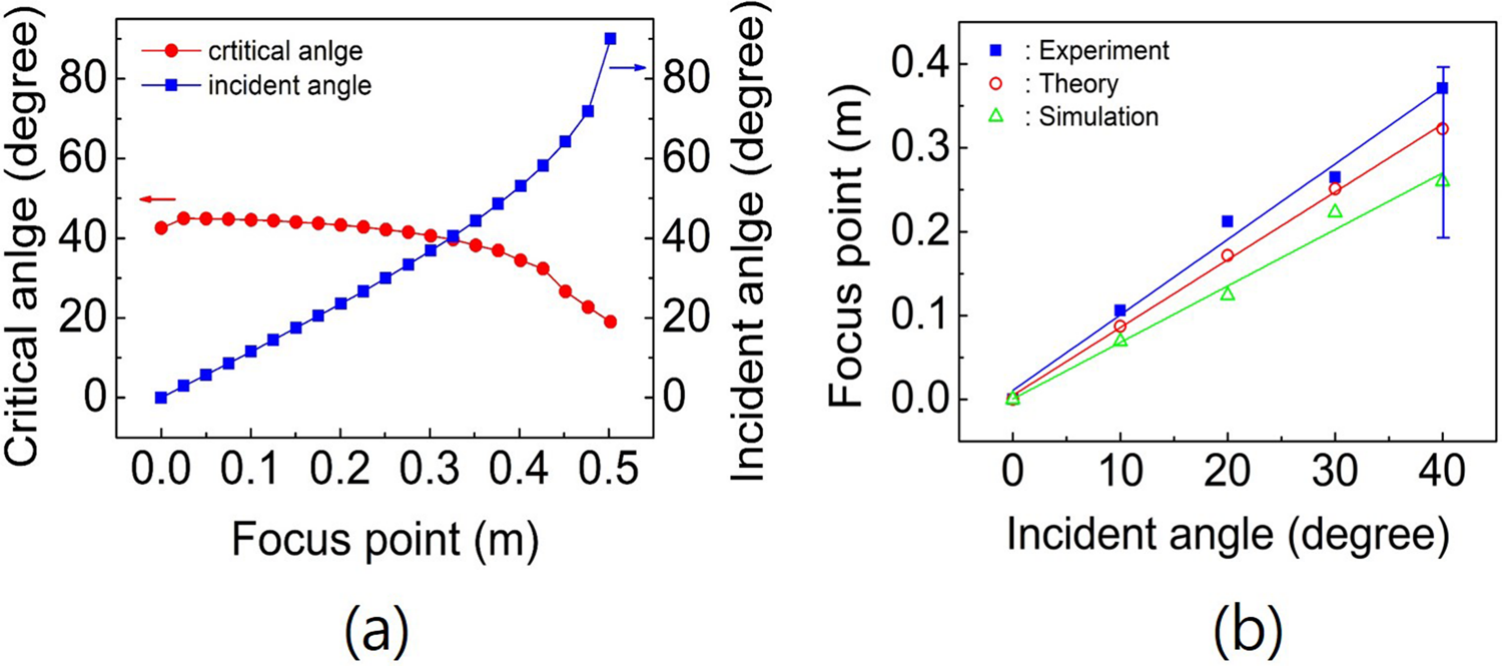
(**a**) shows the distribution of the critical angle (red circle) according to the refractive index distribution of the flattened surface and the theoretical focus point (blue square) on the flattened surface for the angle of incidence. (**b**) shows the focus point on the flattened surface for the angle of incidence. Here, the blue squares, red open circles and green open triangles represent the measured, theoretical and simulated results, respectively. The error bar indicates that the focus point is spread.

Figure 4a–e shows the experimental results of the focusing of acoustic waves with a frequency of 850 Hz after passing through the device for incident angles of 0°, 10°, 20°, 30°, 40°. From the figures, we can observe changes in the displacement of the focus along the surface from the centre of the transformed flattened surface of the hemispherical lens with the incident angles. Since the refractive index values are all different along the points of the flattened surface, as in Fig. 1b, we can consider the distribution of the critical angle along the surface^26,35^. This distribution is plotted with red circles in Fig. 5a. The blue squares show the incidence angles with respect to the theoretical focusing point of the acoustic waves on the flattened surface of the device. If the incident angle is smaller than the critical angle inside the device, some of the acoustic rays are transmitted to the outside, and the rest of the rays are reflected from the flattened surface of the device. If the incidence angle is larger than the critical angle, the acoustic rays cannot transmit and reflect from the surface due to total internal reflection. When the incidence angle is $39.8^{\circ }$, the expected focusing point is displaced along the surface approximately 322.5 mm from the centre of the flattened surface, and the critical angle corresponding to the point is equal to the incidence angle, as shown in Fig. 5a. Therefore, total internal refection occurs at this angle (or at the corresponding point). From this figure, we can expect that focusing occurs only when the incidence angle is smaller than the critical angle, as shown in Fig. 4a–d. When the incidence angle is nearly equal to the critical angle, we observe that the focused acoustic spot spreads without collecting at one point due to the effects related to the total internal reflection of the acoustic waves, such as the evanescent waves on the surface boundary, as shown in Fig. 4e.

For the frequency of 850 Hz, the focus points on the flattened surface with respect to the incident angles are measured and plotted in Fig. 5b. The blue squares, red open circles and green open triangles represent the experimental values, theoretically calculated values and simulated values, respectively. The error bar indicates that the focus is spread. As shown in Fig. 5b, the actual experimental value of the focus point on the flattened plane is always greater than the theoretically expected or the simulated value. Three regressive lines were obtained with a linear fit using the least square method for each group. The slopes of the blue line for the experimental group and the red line for the theoretical group and the green line for the simulated group are $9.01 \times 10^{-3}$ and $8.09 \times 10^{-3}$ and $6.74 \times 10^{-3}\hbox { m/ }^{\circ }$, respectively. Although there is a small but apparently distinguishable difference between first two values $(\simeq 10.8\%)$, there is an important physical phenomenon in the difference: the acoustic GHS by total internal reflection^25,26^. It is known that this shift occurs at the boundary of two media when the incident wave undergoes total internal reflection. The displacement of the focus due to the acoustic GHS is measured to be approximately $3.68 \times 10^{-2}$ m at $\theta _i = 40^{\circ }$ with $f=850\, {\text{Hz}}$. Comparing the result of experiment with that of simulation, it can be seen that the difference is apparent and can be clearly explained by GHS. The displacement of the experimental focus point relative to the simulated focus point is approximately $9.08 \times 10^{-2}$ m at $\theta _i = 40^{\circ }$ with $f=850\, {\text{Hz}}$. In the figure, the simulated value is lower than the theoretical value. It seems to be due to the approximation of the refractive index distribution. The simulation results for the incident angles are shown in the Supplementary Information.

When plane waves are incident on the device from the outside, the angle of incidence of the wave is constant. After entering the device, the waves (or rays) have different directions because they leave from different points on the hemisphere to a certain focus point on the flattened surface^22^. Therefore, the incident acoustic waves at the focal point, which is on the flattened surface, can be thought of as a wave (or ray) collection with an angular distribution of various directions around the incident angle determined from the outside. In these waves converged to the focus point, a portion of the acoustic waves with an angular direction smaller than the critical angle at that point can transmit the boundary surface with different transmittance ratios with respect to the incident angle and rest of them are reflected; however, the waves with an angular direction larger than the critical angle are nearly reflected with the effect of GHS, and parts of the waves propagate as evanescent waves^26,35^. Therefore, due to the angular distribution of the incident waves with respect to the critical angle, the propagation direction of the transmitted waves rotates in the direction of the outward normal. Figure 4f shows the simulation result when the incident angle is $20^{\circ }$ for a comparison with Fig. 4c. The white arrows in the figures show the propagation directions of the acoustic waves after passing through the flattened surface. In the experiment, the propagation direction is rotated $\sim 35^{\circ }$ in the direction of the outward normal when compared to the simulation result. This rotation of the propagation direction shows the effect of acoustic Fresnel filtering, which is similar to the effect in optics^27,28^. We observe that the closer the external incidence angle is to the critical angle, the larger the focus spreading effect. When the angle of incidence significantly exceeds the critical angle ($\theta _{i}\gtrsim 55^{\circ }$), the waves are not transmitted. In this case, the waves with an angular distribution for which the angular component of the incident direction is less than the critical angle are very small or nearly zero.

## Conclusion

With a 2D slice of a 3D hemispherical acoustic Luneburg lens using a quasi-conformal transformation and asymmetric FCOC unit cells, we performed an experiment with a simulation and observed the characteristics of the transformed flattened device in the frequency region that satisfies the homogeneous medium condition of the metamaterial. Using the approximation that the values of $n_{33}$ are nearly equal to the values of $n_{11}$, the desired focusing effect, such as focusing of acoustic plane waves at the antipodal points on the transformed surface of the opposite side of the device with respect to the incident angles and frequencies, was observed. The attenuation losses of the system were measured and compared with those of an untransformed system with respect to frequency. The value of the acoustic GHS was determined by comparing the experimental and theoretical and simulated values of the focus points with respect to the incident angle. The effect of acoustic Fresnel filtering due to the angular distribution of the incident waves at the flat surface boundary was verified by comparing the results of the experiment and the simulation.

## Supplementary information

Supplementary Information.
