# Supplementary material for: Hemispherical acoustic Luneburg lens with the acoustic Goos–Hänchen shift and Fresnel filtering effect
Source: Sci Rep. 2020 Nov 4;10:18991. doi: 10.1038/s41598-020-76111-4 (PMC7643133; doi:10.1038/s41598-020-76111-4)
Supplement: Supplementary file 1 — Supplementary Information. [file 41598_2020_76111_MOESM1_ESM.pdf]

Hemispherical acoustic Luneburg lens with the acoustic Goos-Hänchen shift and Fresnel filtering effect: Supplementary Information

Choon Mahn Park[1], Geo-Su Yim[2], Kyuman Cho[3], and Sang Hun Lee[3,*]

[1]Dong-A University, Department of Materials Physics, Busan, 49315, South Korea
[2]Pai Chai University, Department of Electrical Engineering, Daejeon, 35345, South Korea
[3]Sogang University, Department of Physics, Seoul, 04107, South Korea
[*]linuet@naver.com

ABSTRACT

In this supplementary information, we introduce the effective mass density of the system and simulation results.

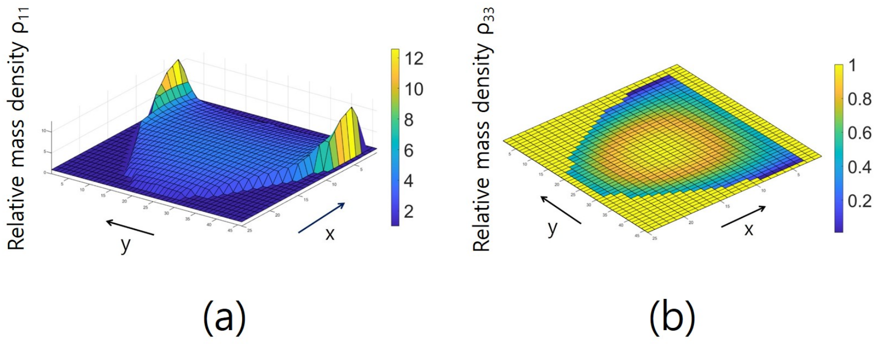

Figure S1. (a) and (b) show the distribution of the relative mass density ρ_{11} and ρ_{33} corresponding to the refractive indices n_{11} and n_{33}, respectively. Here, the relative bulk modulus is set to constant 1. In figure (a), the outermost largest value was not drawn.

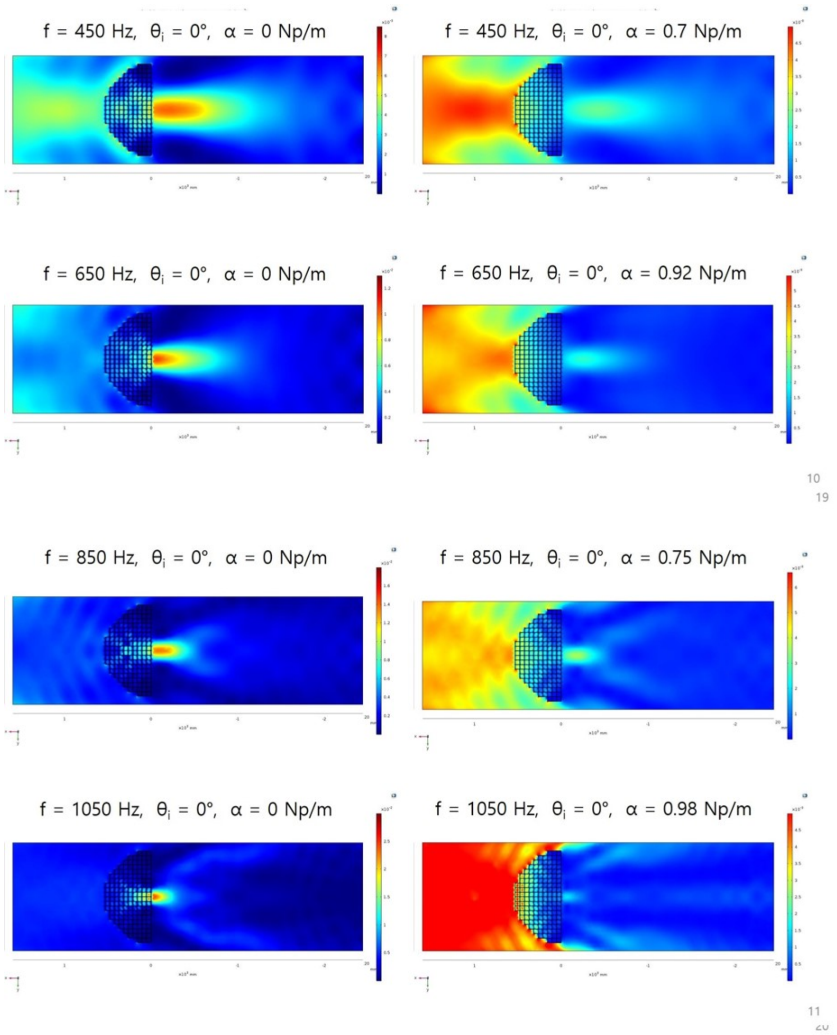

Figure S2. Simulation results with frequency. The figures in the left column are the results without taking into account the attenuation losses. The figures in the right column show the results of considering the attenuation losses. The intensity range of each color bar of the figures has arbitrary values in each figure.

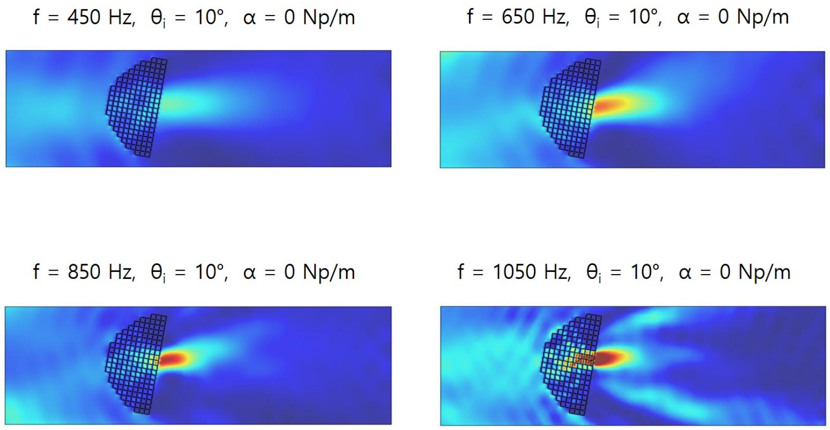

Figure S3-a. Simulation results with frequency when the incident angle is 10°, where the attenuation loss is not considered. The intensity range of each color of the figures has arbitrary values in each figure.

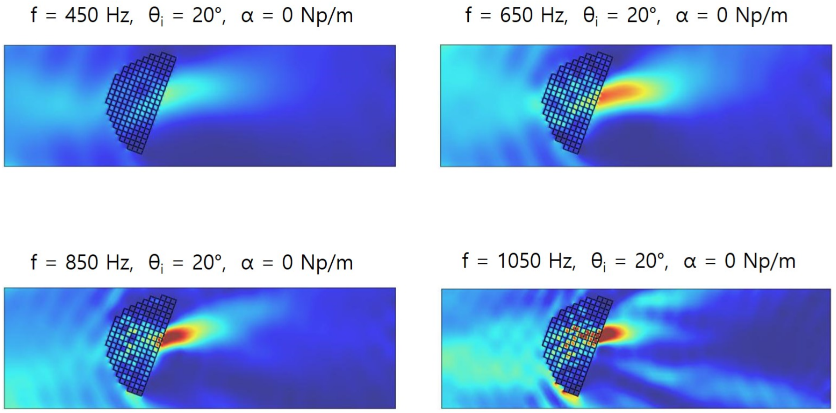

Figure S3-b. Simulation results with frequency when the incident angle is 20°, where the attenuation loss is not considered. The intensity range of each color of the figures has arbitrary values in each figure.

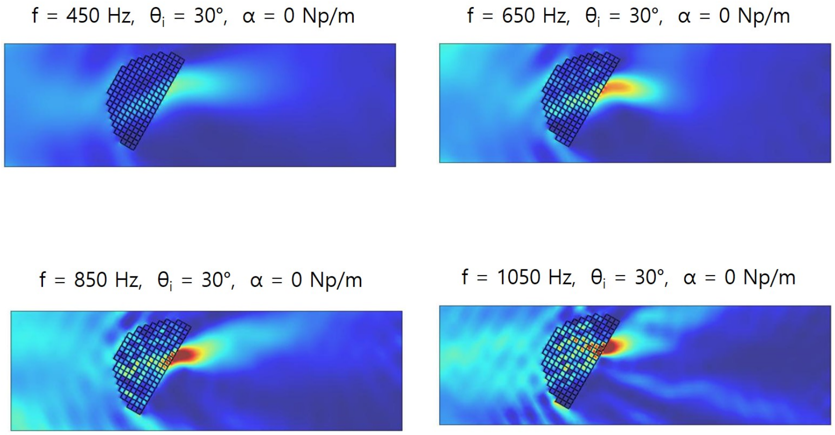

Figure S3-c. Simulation results with frequency when the incident angle is 30°, where the attenuation loss is not considered. The intensity range of each color of the figures has arbitrary values in each figure.

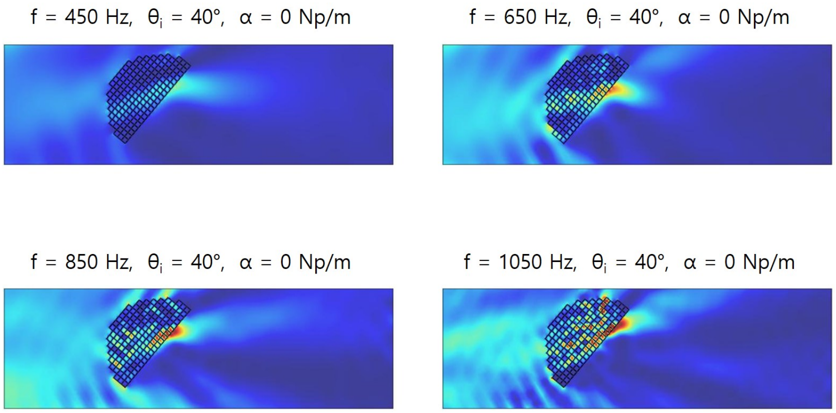

Figure S3-d. Simulation results with frequency when the incident angle is 40°, where the attenuation loss is not considered. The intensity range of each color of the figures has arbitrary values in each figure.

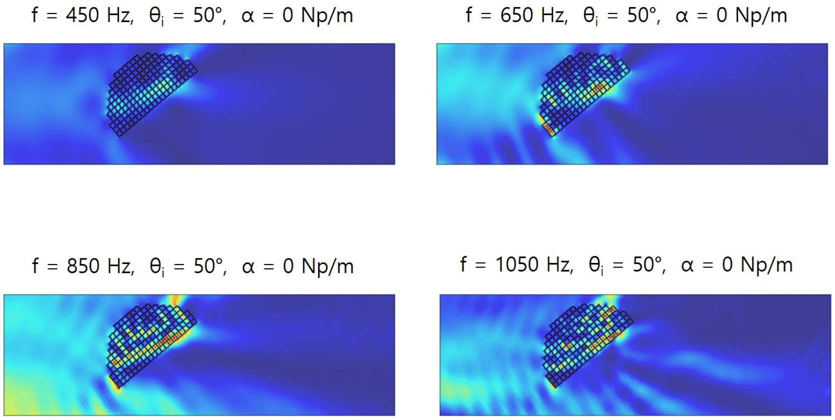

Figure S3-e. Simulation results with frequency when the incident angle is 50°, where the attenuation loss is not considered. The intensity range of each color of the figures has arbitrary values in each figure.
